# Evolutionary innovation and conservation in the embryonic derivation of the vertebrate skull

**DOI:** 10.1038/ncomms6661

**Published:** 2014-12-01

**Authors:** Nadine Piekarski, Joshua B. Gross, James Hanken

**Affiliations:** 1 Department of Organismic and Evolutionary Biology, Museum of Comparative Zoology, Harvard University, 26 Oxford Street, Cambridge, Massachusetts 02138, USA

## Abstract

Development of the vertebrate skull has been studied intensively for more than 150 years, yet many essential features remain unresolved. One such feature is the extent to which embryonic derivation of individual bones is evolutionarily conserved or labile. We perform long-term fate mapping using GFP-transgenic axolotl and *Xenopus laevis* to document the contribution of individual cranial neural crest streams to the osteocranium in these amphibians. Here we show that the axolotl pattern is strikingly similar to that in amniotes; it likely represents the ancestral condition for tetrapods. Unexpectedly, the pattern in *Xenopus* is much different; it may constitute a unique condition that evolved after anurans diverged from other amphibians. Such changes reveal an unappreciated relation between life history evolution and cranial development and exemplify ‘developmental system drift’, in which interspecific divergence in developmental processes that underlie homologous characters occurs with little or no concomitant change in the adult phenotype.

## Abstract (web summary)

It is unclear whether the embryonic origin of skull bones is evolutionarily conserved. Here, the authors show that the pattern of cranial development of the Mexican axolotl is similar to that reported for other vertebrates, but the pattern in the African clawed frog, another amphibian, is unique.

## Graphical abstract

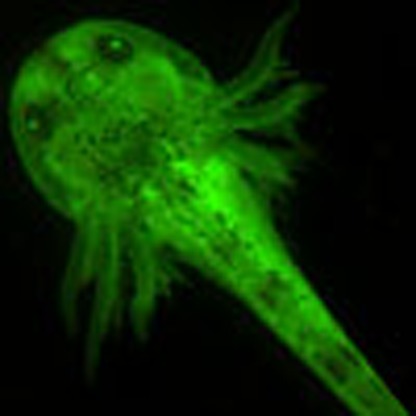

Evolutionary change in the morphology of the bony skull, or osteocranium, underlies every major adaptive transition in vertebrate history^1^. Its developmental basis has been a subject of intense study for more than 150 years, yet many essential features remain unexplored in most taxa. A key unresolved issue, but one central to gaining an understanding of the underlying genetic and developmental mechanisms of craniofacial patterning, concerns the extent to which embryonic derivation of individual bones is evolutionarily conserved or labile^2^. It is generally assumed that the pattern of embryonic origin of skull bones is highly conserved among vertebrates, but data from key groups, such as amphibians, are lacking. We performed long-term fate mapping using green fluorescent protein (GFP)-transgenic Mexican axolotl (*Ambystoma mexicanum*) and African clawed frog (*Xenopus laevis*) to document the contribution of individual cranial neural crest (CNC) streams to the adult osteocranium in these two amphibian species. Here we show that the axolotl pattern is strikingly similar to that reported in amniotes; it likely represents the ancestral condition for tetrapods. Unexpectedly, we also show that the pattern in *Xenopus* is much different from that observed in all other vertebrates studied to date, including the axolotl. The pattern in *Xenopus* constitutes a unique, derived condition that evolved after the anuran clade diverged from other living amphibians, possibly in association with the extreme metamorphosis characteristic of frogs. Embryonic derivation of the bony skull, while highly conserved among many species, exhibits extensive evolutionary innovation in at least one conspicuous vertebrate lineage. Such changes exemplify the phenomenon of ‘developmental system drift’, in which interspecific divergence in developmental processes that underlie homologous characters occurs with little or no concomitant change in the resulting adult phenotype^3^.

Detailed comparisons of two amniote models, the domestic chicken and the house mouse, reveal striking similarity in the relative contributions of two embryonic cell populations, CNC and paraxial mesoderm, which populate discrete and largely non-overlapping territories in the skull^4^,^5^,^6^,^7^. A similar pattern of CNC contribution to the craniofacial skeleton has been reported in zebrafish, a distant phylogenetic relative^8^,^9^ (Fig. 1). Such observations support claims that patterns of embryonic derivation of vertebrate cranial tissues as determined by the neural crest, including the neural crest–mesoderm interface, are largely, if not completely, conserved during vertebrate evolution^10^,^11^,^12^. This, in turn, implies that neither changes in the relative contributions of neural crest and mesoderm nor changes in their specific cartilaginous or bony derivatives underlie major evolutionary changes of skull form. Yet, such claims rely on a limited sampling of vertebrate diversity and do not include phylogenetically critical groups, such as amphibians, which represent a key transitional stage in evolution from bony fishes to amniotes^2^,^12^. Another compelling feature of amphibians is the presence in many species of discrete larval and adult life history stages, each with a distinctive cranial morphology. Whereas in salamanders the metamorphic transition from larva to adult is modest and gradual, in anurans it is extensive and abrupt^13^,^14^. In frogs, for example, bones do not begin to differentiate until metamorphosis, when they largely replace an exclusively cartilaginous larval skull^15^,^16^. This is unlike most other vertebrates in which bones typically form in the embryo. The consequences of a biphasic ontogeny and postembryonic metamorphosis for the embryonic derivation of the adult cranium are largely unknown.

Here we use transgenic strains of Mexican axolotl (*A. mexicanum*; ref. 17) and African clawed frog (*X. laevis*; ref. 18), each representing a separate order of amphibians, to map the contribution of CNC to the bony adult skull in each species. The timing and extent of cranial metamorphosis in *Xenopus* is typical of anurans generally^15^,^16^, and while the adult axolotl retains a larva-like external morphology, it nevertheless forms many of the skull bones found in metamorphosing urodeles^14^. Extensive contribution of CNC to the cartilaginous larval skull has been demonstrated in several amphibian species through the use of a variety of extirpation and vital-labelling procedures^19^. Comparable data regarding the embryonic derivation of the bony skull, however, has been extremely difficult to obtain because of the absence of a reliable and permanent cell marker that can be applied to neural crest cells in the early embryo and effectively label adult derivatives, such as bone, that do not form until after hatching or even weeks or months later, after metamorphosis. Our transgenic labelling protocol overcomes these technical challenges posed by the metamorphic ontogeny and its extended time interval between embryo and adult^17^,^18^. By grafting GFP-expressing cells from transgenic donor embryos into wild-type hosts, we are able to evaluate the relative contributions from all three migratory streams of CNC to each bone in the adult skull, including both intramembranous and endochondral elements.

We find that the pattern of CNC derivation of the bony skull in the axolotl, in which nearly the entire CNC contribution derives from the mandibular migratory stream, is strikingly similar to that reported in amniotes. This pattern may represent the ancestral condition for tetrapods, and possibly even bony fishes, which is retained in most extant clades. The pattern in *Xenopus*, however, is very different. There are substantial contributions to the skull from all three CNC streams, including derivation of a portion of the upper jaw from the hyoid stream instead of the mandibular stream, which is the typical source of the vertebrate jaw skeleton. This pattern likely evolved after anurans diverged from other living amphibians, possibly in association with the extreme cranial metamorphosis characteristic of frogs. The combination of evolutionary conservation and innovation seen in these features of cranial development constitutes an instance of developmental system drift. It mandates a more careful and nuanced use of ontogenetic data as a criterion for evaluating the homology of skull bones among vertebrates than has been done previously, at least in some comparisons. Indeed, some of the most widely accepted homologies, particularly those involving the skull vault in tetrapods, are likely incorrect and require reevaluation.

## Results

### CNC derivation of the skull in axolotl resembles amniotes

**Figure 1:**
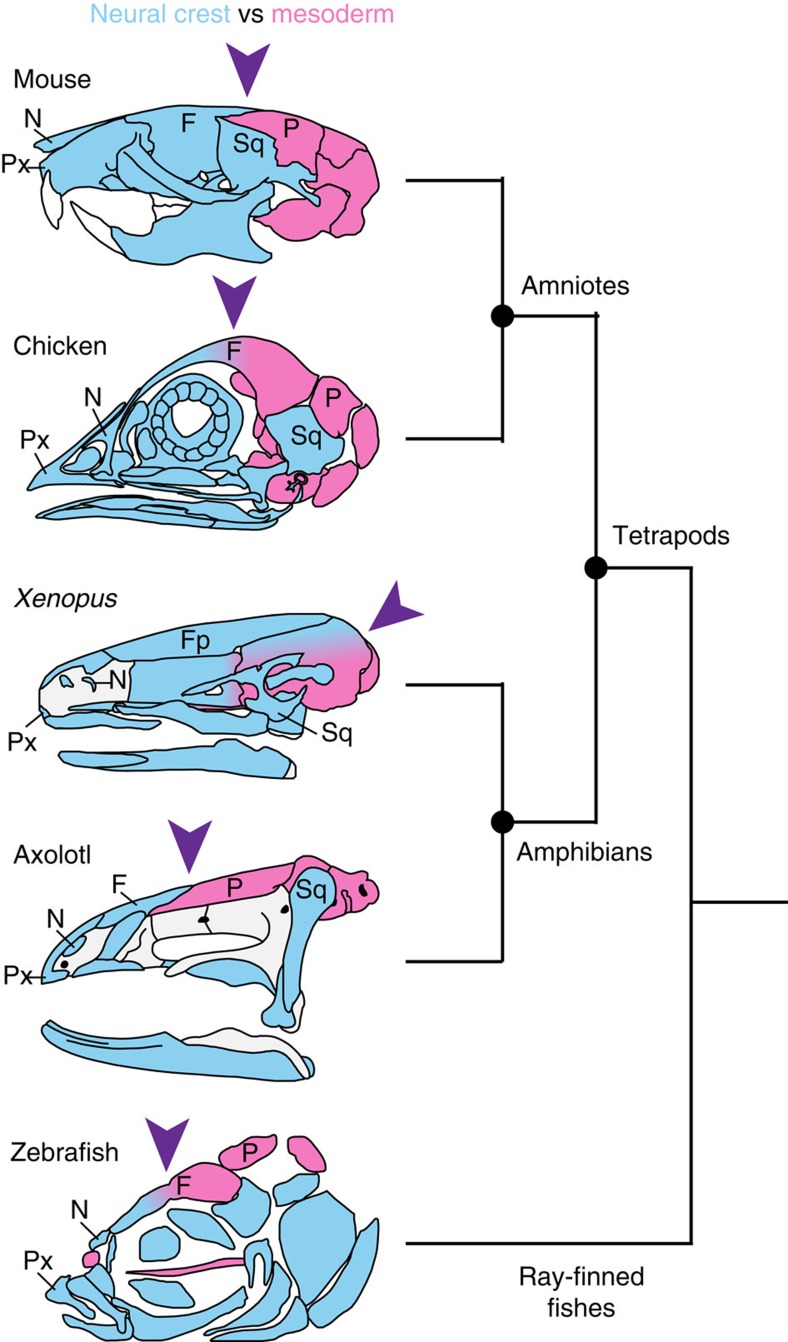
Embryonic origin of the bony skull in five vertebrate model organisms arrayed on a simplified vertebrate phylogeny. Neural crest-derived territories (blue) have been verified experimentally in each species, although the specific contributions from individual migratory streams are reported only for chicken, axolotl and *Xenopus*. Derivation of remaining components from mesoderm (magenta) has been verified experimentally in mouse and chicken and is presumed for the remaining species. Arrowheads point to the neural crest–mesoderm interface in the skull roof, which is displaced caudally in *Xenopus*. Data for zebrafish are from refs 8, 9; diagram is based on ref. 8 (figure reproduced with permission from PLoS). Data for axolotl and *Xenopus* are from the present study; skulls are redrawn from refs 16, 42, respectively (figures reproduced with permission from John Wiley and Sons). Data for chicken are from ref. 43; diagram is based on ref. 44 (figure reproduced with permission from John Wiley and Sons). Data for mouse are from refs 7, 45; diagram is based on refs 4, 46 (figure reproduced with permission from John Wiley and Sons). F, frontal; Fp, frontoparietal; N, nasal; P, parietal; Px, premaxilla; Sq, squamosal.

**Figure 2:**
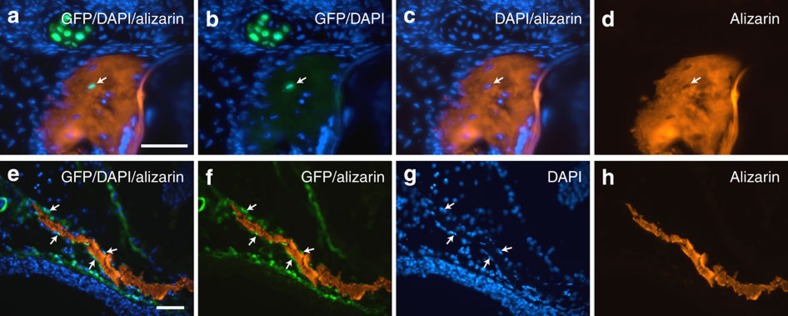
Transverse sections through an axolotl skull showing GFP labelling of bone. GFP-labelled cells are rarely seen within the bony matrix, which is largely acellular (upper row), but they are abundant in the periosteum, a connective tissue layer that invests individual bones (lower row). (**a**–**d**) A single GFP-labelled osteocyte (arrow) in the bony matrix of the premaxilla. (**e**–**h**) Four labelled cells (arrows) in the periosteum of the parasphenoid. In each row, a single section is depicted four times at the same magnification, each with a different combination of fluorescent illumination. Labelling: DAPI-stained nuclei (blue); GFP-positive cells (green); and alizarin-stained bone matrix (red). Scale bar, 100 μm.

**Figure 3:**
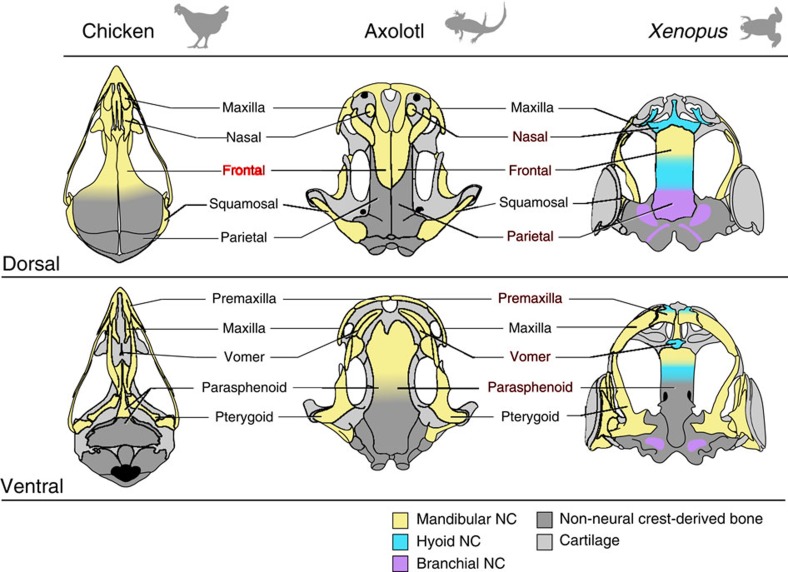
Patterns of CNC derivation of the bony skull differ between *Xenopus* and axolotl. Coloured regions denote contributions from individual migratory streams of CNC. Red labels denote homologous bones that have a different embryonic origin between species. Data for axolotl and *Xenopus*, two amphibians, are from the present study; skulls are redrawn from refs 16, 42, respectively. Data for the domestic chicken, an amniote, are from ref. 43; diagram is based on ref. 44 (figure reproduced with permission from John Wiley and Sons).

**Figure 4:**
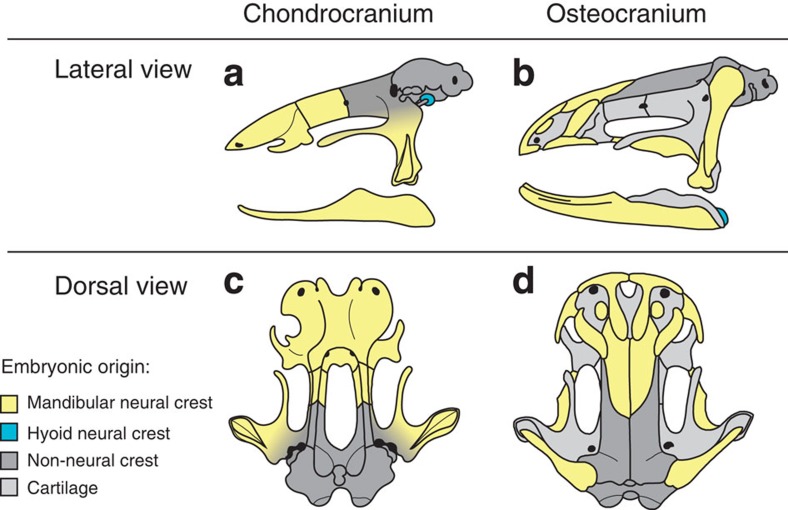
CNC derivation of cartilages and bones in the skull of the adult axolotl. Most cartilages (**a**,**c**) and bones (**b**,**d**) are derived from the mandibular stream (yellow). Hyoid stream contributions (blue) are limited to (**a**) the stapes of the middle ear and (**b**) the retroarticular process of the lower jaw. There is no contribution to the skull proper from the branchial stream, which contributes extensively to the branchial or gill skeleton (not illustrated). The remainder of the skull (dark grey) is presumably derived from paraxial mesoderm, although this remains to be confirmed experimentally. Skulls are redrawn from ref. 42.

**Figure 5:**
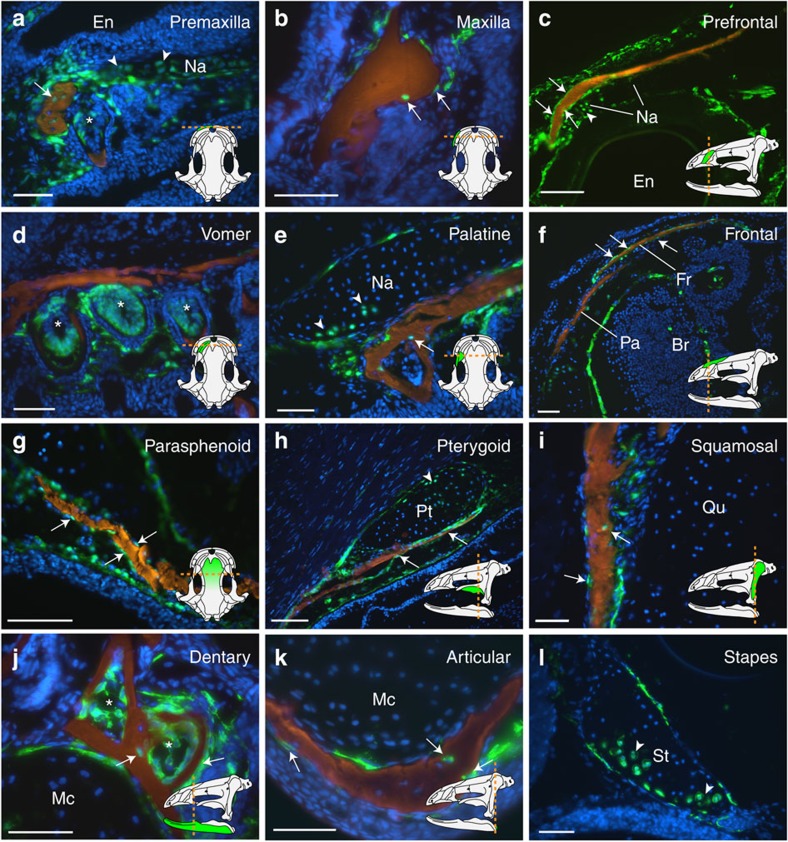
Mandibular stream neural crest is the principal source of skull bones in the axolotl. Panels depict transverse sections from juvenile axolotls that received embryonic grafts of mandibular (**a**–**j**) or hyoid (**k**,**l**) stream neural crest. Schematics of skulls show bone of interest (green); dashed red lines indicate plane of section. GFP-labelled cells are green; bony matrix is stained red; and cell nuclei are counterstained blue (except **c**). Arrows point to labelled osteocytes within bony matrix or labelled periosteal cells. Chondrocytes (arrowheads) and mesenchymal core of teeth (*) are also labelled. Br, brain; En, external naris; Fr, frontal; Mc, Meckel’s cartilage; Na, nasal cartilage; Pa, parietal; Pt, pterygoid cartilage; Qu, quadrate; St, stapes. Scale bar, 100 μm.

Even after rearing times as long as 8 months, GFP-expressing cells derived from grafts prominently label cranial osteocytes. Such labelling is typically confined to the periosteum, a connective tissue layer that invests individual bones, and is rarely seen within the bony matrix, which is largely acellular (Fig. 2). In the axolotl, CNC contributes to an extensive anterior portion of the osteocranium, including the premaxilla, maxilla, nasal, frontal, vomer and the anterior portion of the parasphenoid, as well as the squamosal and pterygoid bones laterally and ventrally and the entire lower jaw (Figs 1 and 3, 4, 5; Table 1). Except for the posterior tip (retroarticular process) of the articular bone in the lower jaw, which is derived from the hyoid neural crest stream, the entire CNC contribution to the bony skull derives from the mandibular stream (hyoid neural crest cells also contribute to the cartilaginous stapes; Figs 4 and 5l). In the skull roof, GFP labelling is found throughout the frontal bone, but there is no indication of any CNC contribution to the parietal bone, which articulates with the frontal posteriorly. GFP-expressing cells, however, are visible deep to the anterior portion of the parietal bone, where they label meninges that invest the underlying brain (Fig. 5f). This pattern of derivation of the osteocranium mirrors that seen in the chondrocranium, in which the mandibular stream is the nearly exclusive source of CNC-derived cranial cartilages anteriorly and ventrally and a CNC contribution is largely absent posteriorly (Fig. 4).

### CNC derivation of the skull in *Xenopus* is unique

**Figure 6:**
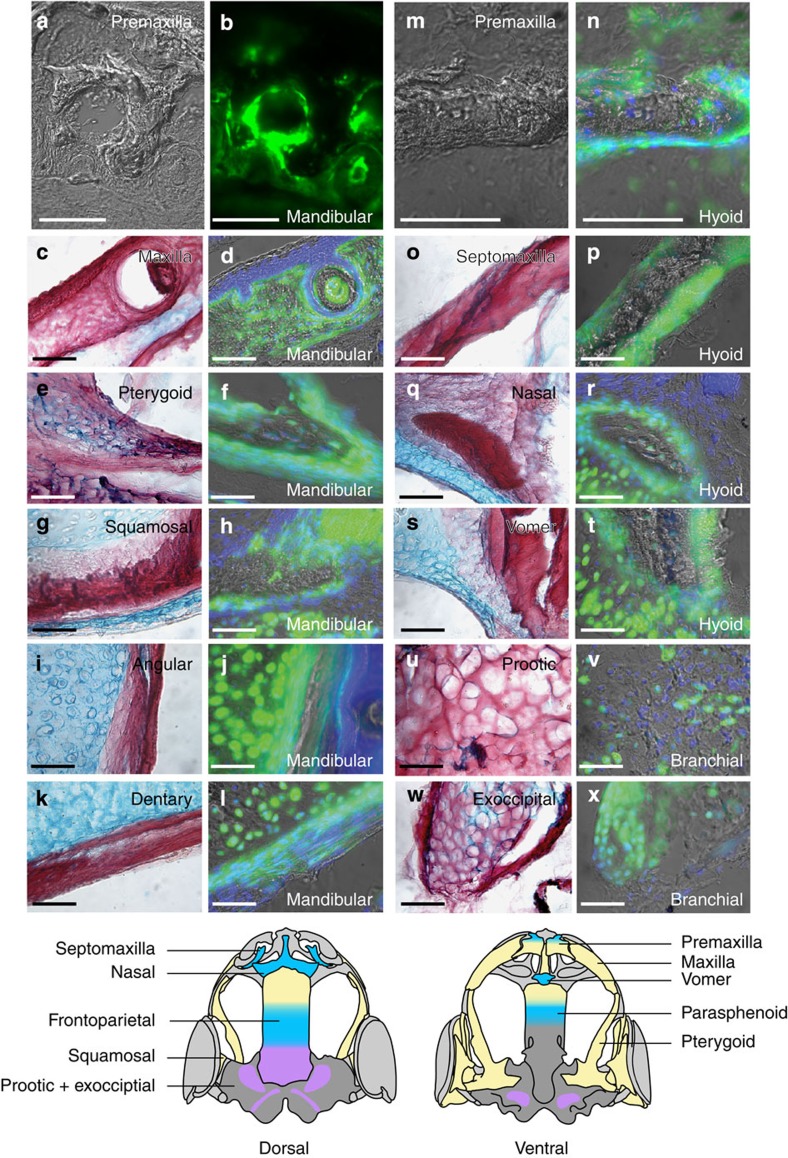
All three CNC streams contribute to the osteocranium in *X. laevis*. Transverse sections are from postmetamorphic frogs that received GFP-positive embryonic grafts of the mandibular (**a**–**l**), hyoid (**m**–**t**) or branchial (**u**–**x**) stream. Each pair of images depicts adjacent sections of the grafted (left) side. In most, the left section is stained histologically to reveal cartilage (blue) and bone (red); **a**,**m** are viewed with Nomarski (differential interference contrast) microscopy. The right section is immunostained for GFP (green); cell nuclei are counterstained blue. Note the composite origin of the premaxilla from both mandibular (**a**,**b**) and hyoid (**m**,**n**) streams. Scale bar, 50 μm.

The pattern of CNC contribution to the osteocranium in *Xenopus* is very different. Overall, the crest-derived territory is extensive, it incorporates most of the bony skull, including portions of the otic region caudally, which receives no CNC contribution in the axolotl^20^ (Figs 1, 3 and 6; Table 1). Non-crest-derived regions are confined to the anterolateral portion of the prootic and to posterior portions of the fused parasphenoid–sphenethmoid and the exoccipital. Whereas most bones are derived each from a single neural crest stream, three adult bones receive contributions from two (premaxilla and parasphenoid–sphenethmoid) or even three (frontoparietal) adjacent streams. Moreover, there are substantial contributions from both hyoid and branchial streams. Perhaps, the most unusual feature is the unique derivation of all or part of several rostral bones associated with the upper jaw from the hyoid stream instead of the mandibular stream, which populates the first oropharyngeal arch and is the typical source of the vertebrate jaw skeleton^21^. This pattern yields the unprecedented, reversed rostrocaudal sequence, visible both dorsally and ventrally and involving both adult bones and adult cartilages^22^, in which the rostral-most region of the postmetamorphic skull is derived from the hyoid stream, followed caudally by derivatives of the mandibular stream, then additional derivatives of the hyoid stream and finally by derivatives of the branchial stream (Fig. 3). Interestingly, the reversed sequence is not seen in the larval skull, which instead displays the typical sequence of mandibular stream-derived cartilages rostrally, followed by hyoid stream cartilages and finally branchial stream cartilages caudally (Supplementary Fig. 1; ref. 23).

## Discussion

Our data bolster claims that the embryonic origin of the skull is in general highly conserved evolutionarily among tetrapods: the pattern of CNC contributions to the bony skull in the axolotl, an amphibian, closely resembles that reported for amniotes (Figs 1 and 3). At the same time, our data reveal a surprising deviation from that conserved pattern in *X. laevis*, another amphibian: the embryonic derivation of several bones in *Xenopus* differs from that of homologous bones in both the axolotl and amniotes. For example, both the nasal and the vomer in axolotl and chicken are derived from mandibular stream neural crest, whereas in *Xenopus*, each bone receives cellular contributions from the hyoid stream. The parietal bone is derived from neural crest in *Xenopus* but is not derived from neural crest in the other species.

**Figure 7:**
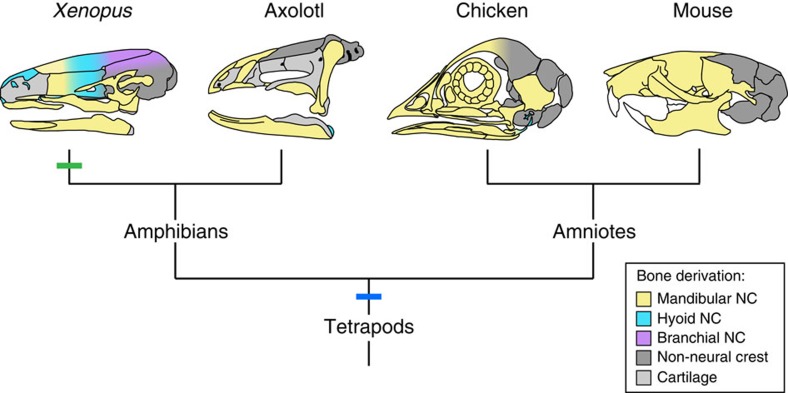
Hypothesis for the evolution of CNC derivation of the bony skull. Coloured regions depict contributions to the osteocranium from the three CNC migratory streams in four tetrapod model systems. Stream-level contributions are not known in the mouse, but they are presumed to resemble those in the chicken^46^. It is most parsimonious to posit that urodeles and amniotes share a common pattern of CNC derivation, which evolved no later than their common tetrapod ancestor (blue bar on the simplified phylogeny), and that the unique pattern in *Xenopus* evolved after the anuran clade diverged from urodeles (green bar).

On the basis of these data, we suggest a novel hypothesis for the evolution of embryonic derivation of the vertebrate skull (Fig. 7). We propose that urodeles and amniotes share an identical pattern of CNC derivation of the osteocranium, which evolved in their common tetrapod ancestor, if not earlier, and is retained in most extant clades. We further propose that the unique pattern of CNC derivation of the osteocranium in *Xenopus* evolved after the anuran clade diverged from urodeles and in association with the extreme, biphasic skeletal ontogeny characteristic of most frogs. Metamorphic remodelling of the skull in anurans is extensive and abrupt, especially anteriorly^13^,^14^; principal changes include resorption of numerous larval-specific cartilages and *de novo* formation of adult-specific cartilages and all bones. The unusual pattern seen in *Xenopus* may be a consequence of these dramatic morphogenetic rearrangements and the substantial delay in the onset of ossification, which in metamorphosing frogs is an exclusively postembryonic phenomenon^15^.

Additional data are needed to more precisely resolve the phylogenetic distribution of these two patterns. The presence of a similar pattern of CNC derivation of the osteocranium in zebrafish^8^,^9^ suggests that the urodele/amniote pattern may represent the ancestral condition for tetrapods, and possibly even bony fishes. Such a broad comparison is complicated, however, by the uncertain homologies between several skull bones in tetrapods and their presumed counterparts in zebrafish and other ray-finned fishes^24^, and by the lack of data regarding the CNC derivation of skull bones in zebrafish at the level of individual migratory streams. Conversely, *Xenopus* and its close phylogenetic relatives exhibit several unusual features of both embryonic development and larval and adult morphology, which are not shared with other frogs, let alone other vertebrates^16^,^25^,^26^,^27^. The fact that in *Xenopus* the same two features that define its unique pattern of adult osteocranial development—a substantial contribution from the hyoid CNC stream, and reversal of the sequence of derivation of rostral elements—also characterize embryonic derivation of adult cranial cartilages^22^ (Supplementary Fig. 2), and that both features are absent from the cartilaginous larval skull^23^, supports the idea of a mechanistic link between CNC derivation and cranial metamorphosis. Yet, a substantial contribution from the hyoid CNC stream to the cartilaginous larval neurocranium in *Bombina orientalis*^28^, another frog, suggests that the pattern in *Xenopus* may not be characteristic of anurans generally and that this one tetrapod clade instead may harbour substantial interspecific variation in fundamental features of cranial development.

The strikingly similar pattern of neural crest derivation of the osteocranium that is shared by the axolotl and amniotes may reflect the existence of phylogenetically ancient constraints on cranial development in vertebrates. Yet, the presence of a dramatically different, unique pattern of derivation in *Xenopus* indicates that such constraints may be circumvented in individual lineages. Embryonic derivation of the skull thus is both highly conserved and evolutionary labile, a characterization that also extends to individual homologous bones, as traditionally defined. Recent comparative studies provide abundant evidence that homologous morphological characters, whose similarity is due to common ancestry, may form via different developmental and genetic pathways in different species^29^,^30^. Indeed, such interspecific divergence in underlying developmental processes may occur with little or no concomitant change in the resulting adult phenotype, a phenomenon termed ‘developmental system drift’^3^,^31^,^32^. Similarly, both embryonic neural crest and mesoderm are capable of contributing to the same skull bones following experimental manipulation^11^. Our results exemplify these phenomena and caution against the use of ontogenetic data as an exclusive or infallible criterion for evaluating the homology of skull bones among vertebrates. Remarkably, this message was articulated more than 75 years ago by the renowned comparative embryologist Gavin de Beer^33^, well before the advent of molecular genetics, transgenesis and the wide array of sophisticated experimental and analytical tools that are available to researchers today. At the same time, our data suggest that some widely accepted homologies for skull bones among tetrapods, particularly those involving the skull vault (frontal, parietal and so on), may be incorrect in at least some taxa and in this way obscure, rather than reveal, important trends in comparative osteology and vertebrate evolution.

## Methods

### Embryonic grafting of CNC

**Figure 8:**
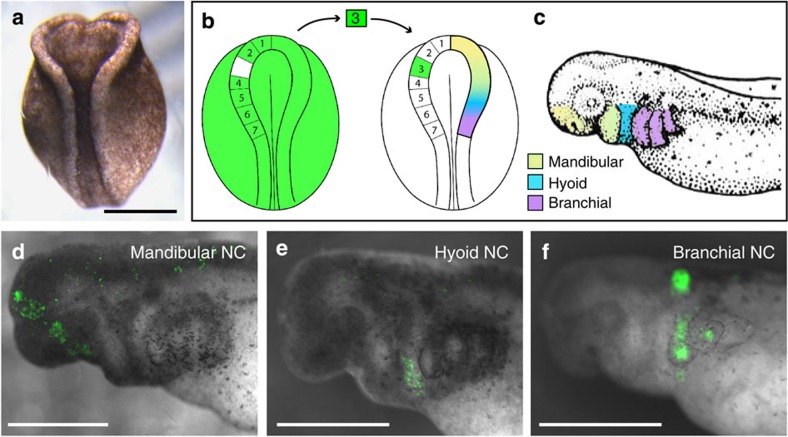
Grafting procedure. (**a**) Photograph of a living stage-16 axolotl embryo^38^, dorsal view, anterior at the top. Paired neural folds are about to meet in the midline and fuse postcranially, but they remain prominent and far apart in the head. (**b**) Drawing of stage-17 embryos depicting the seven regions within the left cranial neural fold^39^ that were grafted individually from GFP-positive donor embryos (green) into wild-type hosts. The approximate locations of premigratory mandibular, hyoid and branchial stream neural crest are depicted on the right side of the host embryo. (**c**) Stage-36 embryo in lateral view depicting migratory streams of mandibular, hyoid and branchial neural crest, which occupy the rostral region of the head and the oropharyngeal arches. (**d**–**f**) Donor-derived CNC cells (green) migrating within the first, second and posterior oropharyngeal arches are visible in living chimeric embryos following grafts of premigratory mandibular, hyoid and branchial stream neural crest, respectively. Mandibular stream neural crest also populates the rostral region of the head in **d**. Lateral views, anterior is to the left. Scale bar, 1 mm.

Grafting experiments to assess the contribution of CNC to the bony skull were performed separately in the Mexican axolotl (*A. mexicanum*) and the African clawed frog (*X. laevis*). We employed transgenic lines of axolotl and *Xenopus* that ubiquitously express GFP and that have been successfully used for long-term fate mapping^17^,^18^,^20^,^22^. In general, segments of mandibular, hyoid or branchial CNC were transplanted from GFP-positive donor embryos into stage-matched, wild-type hosts (Fig. 8). All grafts were performed on the left side; the intact right side served as an internal control. The experimental technique for *Xenopus* is described in several publications; these and associated studies validate our methods for labelling and grafting CNC^22^,^34^,^35^,^36^ (Supplementary Fig. 1). For axolotl, embryos were obtained from the Hanken laboratory breeding colony at Harvard University and from the Ambystoma Genetic Stock Center at the University of Kentucky. In preparation for grafting, the jelly coat was manually removed during late gastrula stages by using watchmaker forceps. The following two sets of transplantation experiments were performed with axolotls. Each experimentally produced chimera was given a unique number and raised individually.

Neural fold transplantations were carried out at neurula stages 15–19 (refs 37, 38), but mostly at stages 16–17, before the paired neural folds have fused in the midline. The neural fold was artificially divided into seven rostrocaudal segments^39^ (Fig. 8b). A small block of dorsal neural fold of the wild-type host embryo was removed by using tungsten needles and replaced with a similar-sized block from the corresponding region of a stage-matched, GFP-transgenic donor. The resulting chimera was assessed over the next few days to confirm which neural crest stream or oropharyngeal arch contained GFP-positive cells (Fig. 8d–f).

CNC stream transplantations were performed at stages 20–25. Here, the cranial epidermis was cut and partly folded back to reveal the underlying CNC streams. Neural crest cells have a dark pigmentation and are easily distinguished from the underlying, lighter mesoderm. A segment of the mandibular, hyoid or branchial neural crest stream was removed from the GFP-negative host embryo and replaced by a comparable segment from the corresponding stream of a GFP-positive donor. In younger embryos, before CNC migration was far advanced, the transplant was taken from the neural tube and thus contained one neural crest stream and a portion of the underlying neural tube. After the transplant was in place, the overlying epidermis was unfolded and held in its original position with a small piece of coverslip glass. The grafted site typically healed within 30 min following surgery. Subsequent migration of GFP-positive cells was documented over the next several days by regular, brief examination with fluorescence illumination as described above.

Chimeric axolotl and *Xenopus* were reared for as long as 8 months, by which time most skull bones had developed, and staged^37^,^40^.

### Histological processing and immunostaining

Infiltration with optimal cutting temperature cryomedium (OCT; Tissue Tek, Sakura Finetek, Tokyo, Japan) was achieved by sequential immersion in 15% sucrose, 30% sucrose, equal parts 30% sucrose and OCT and pure OCT; each step lasted until the specimen sank to the bottom of its container. Specimens were embedded in plastic moulds containing OCT, quick frozen and stored at −80 °C. Serial transverse sections (16–20 μm) were collected onto VWR Superfrost Plus micro slides and stored at −20 °C until further processing.

Antibodies were applied to serial sections to enhance the GFP signal before examination. The primary antibody was omitted occasionally as a control for nonspecific background staining. Sections were rinsed three times for 5 min each in phosphate-buffered saline (PBS; pH 7.4) and in PBST (PBS with 1% Triton X-100). Sections were blocked using 5% normal goat serum in PBST for 2 h at room temperature. The primary antibody against GFP (rabbit polyclonal anti-GFP, ab290; Abcam Antibodies, Cambridge, MA; 1:3,000 in PBST+5% normal goat serum) was applied to the horizontal slides in a humidified chamber overnight at 4 °C. Following a rinse in PBS and immersion for 5 min in PBST, secondary antibody (goat anti-rabbit, Alexa Fluor 488; Life Technologies, Grand Island, NY; 1:1,000 in PBST) was applied to the horizontal slides in a humidified chamber overnight at 4 °C. Following a thorough rinse in PBS, alizarin red S (0.5% in PBS; Sigma Chemicals, Perth, WA) was applied to the horizontal slides for 3 min to stain calcified bone. Subsequently, slides were rinsed in PBS and stained with 4,6-diamidino-2-phenylindole (DAPI; 5 μg ml^−1^; Molecular Probes). Finally, slides were rinsed several times in PBS and mounted with a coverslip using Fluoromount G (Southern Biotech, Birmingham, AL).

### Microscopic examination of sections

GFP labelling of each skull bone was assessed in serial sections from 25 *Xenopus* chimeras that completed metamorphosis and 21 axolotl chimeras. Sections were viewed with a Leica DMRE fluorescent compound microscope (B-filter; Leica, Bannockburn, IL). The intact, unlabelled, right side of each chimera served as an internal control to confirm positive labelling on the left, operated side. Positive labelling was defined as GFP-positive osteocytes and osteoblasts in the bony matrix or GFP-positive cells in the periosteum. To confirm the location of fluorescently labelled cells within the bone in *Xenopus*, sections adjacent to each antibody-stained section were processed with Masson trichrome stain^41^. Adjacent sections in axolotl were stained with alizarin red S (0.5% in PBS; Sigma Chemicals) and DAPI (5 μg ml^−1^; Molecular Probes). Positive labelling in each bone was observed in at least two chimeras.

### Animal care

Animal care procedures are approved by the Harvard University/Faculty of Arts and Sciences Standing Committee on the use of Animals in Research and Teaching. An Animal Welfare Assurance statement is on file with the university’s Office for Laboratory Welfare. Sample sizes represent the minimum numbers of specimens needed to document positive and reproducible labelling in individual bones in the adult skull. Samples were excluded only when they failed to survive to the stage(s) when bones have developed.

## Additional information

**How to cite this article**: Piekarski, N. *et al.* Evolutionary innovation and conservation in the embryonic derivation of the vertebrate skull. *Nat. Commun.* 5:5661 doi: 10.1038/ncomms6661 (2014).

## Supplementary Material

Supplementary Information Supplementary Figures 1-2 and Supplementary References

## Figures and Tables

**Table 1:** CNC derivation of the adult osteocranium in axolotl and *Xenopus* inferred from GFP labelling of individual migratory streams.

**Skull region**	**Skull bones**	
	**Axolotl**	***Xenopus***
Marginal jaw series	Premaxilla*	Premaxilla (pars palatina)*
		Premaxilla (alary process, pars dentalis)†
	Maxilla*	Maxilla*
	—	Septomaxilla†
	Nasal*	Nasal†
Roofing bone series	Prefrontal*	—
	Frontal*	Frontoparietal (anterior)*‡
	Parietal§	Frontoparietal (intermediate)†
		Frontoparietal (posterior)||
Palatal series	Vomer*	Vomer†
	Palatine*	—
	Pterygoid*	Pterygoid*
	Parasphenoid (anterior)*	Parasphenoid–sphenethmoid (anterior)*¶
	Parasphenoid (posterior)§	Parasphenoid–sphenethmoid (intermediate)†
		Parasphenoid–sphenethmoid (posterior)§
Temporal series	**Orbitosphenoid** (anterior)*	Sphenethmoid—see above
	**Orbitosphenoid** (posterior)§	
	Squamosal*	Squamosal*
	**Quadrate** *	**Quadrate** *
Oto-occipital region	**Occipito-otic** §	**Prootic** (medial)||#
		**Prootic** (lateral)§
		**Exoccipital** (anterior)||
		**Exoccipital** (posterior)§
Lower jaw	Dentary*	Dentary*
	—	Angulosplenial*
	Prearticular*	—
	**Articular** (most)*	—
	**Articular** (retroarticular process)‡	

CNC, cranial neural crest; GFP, green fluorescent protein.

Endochondral (cartilage-replacement) bones are in boldface. Sample size for each observation ranges from 2 to 7 specimens (*Xenopus*) and from 3 to 10 (axolotl). GFP-positive cells were always present only on the grafted (left) side. Data are not presented for the middle ear and hyobranchial/hyolaryngeal skeletons.

^*^Mandibular stream derived.

^†^Hyoid stream derived.

^‡^The single frontoparietal bone in adult *Xenopus* is the presumed homologue of the frontal and parietal bones of urodeles and other tetrapods^47^.

^§^No neural crest contribution.

^||^Branchial stream derived.

^¶^In adult *Xenopus*, the parasphenoid is fused to paired sphenethmoid bones, which form from discrete ossification centres earlier in development^16^.

^#^CNC contribution to the medial portion of the prootic bone was inconsistent and incomplete; both labelled and unlabelled cells were present together within the bony matrix (Fig. 6k).
