# Supplementary material for: Evolutionary innovation and conservation in the embryonic derivation of the vertebrate skull
Source: Nat Commun. 2014 Dec 1;5:5661. doi: 10.1038/ncomms6661 (PMC4251486; doi:10.1038/ncomms6661)
Supplement: Supplementary Information — Supplementary Figures 1-2 and Supplementary References [file ncomms6661-s1.pdf]

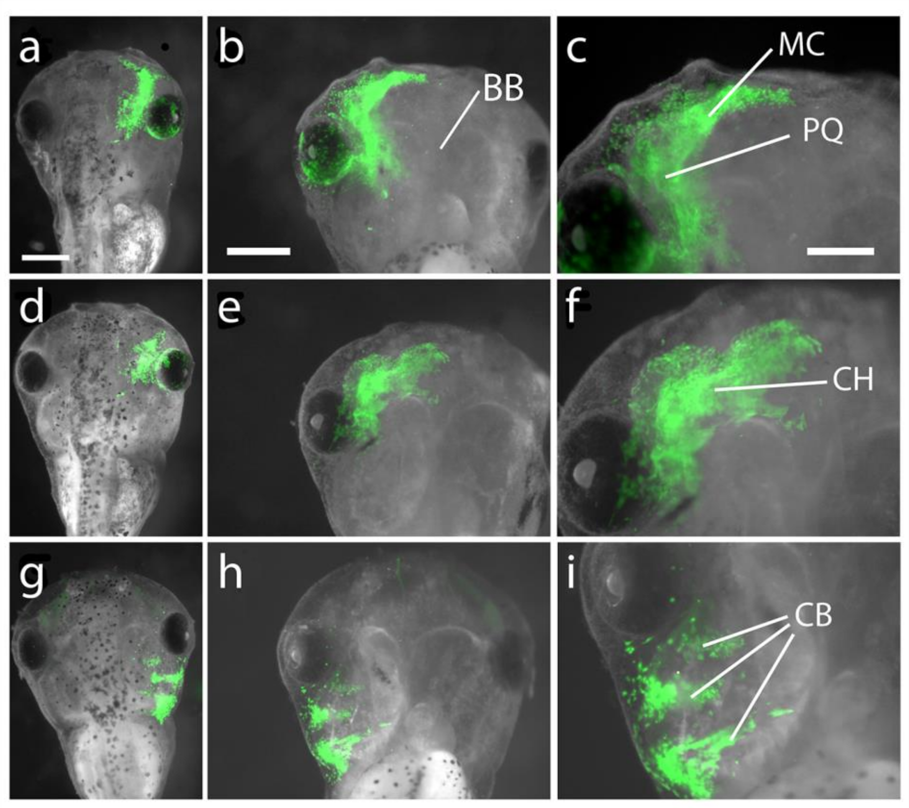

Supplementary Figure 1–Cranial neural crest (CNC) derivation of oropharyngeal cartilages in larval *Xenopus laevis* validates the precision of the grafting procedure used to assess stream-by-stream contributions to skull bones in adults. The same procedure was used to graft explants of premigratory CNC streams from fluorescein dextran-labeled donor embryos[1] into unlabeled hosts. The contribution from each crest stream to individual cartilages was then examined in chimeras at approximately 7 d post-fertilization. These results are the same as those from an earlier study that utilized a different labeling procedure[2]: Meckel's and palatoquadrate cartilages from the mandibular stream (**a–c;** MC and PQ, respectively); ceratohyal cartilage from the hyoid stream (**d–f;** CH); and ceratobranchial cartilages from the branchial stream (**g–i;** CB). Similarly, we found no evidence of CNC contribution to the midline basibranchial cartilage (**b;** BB). Each row features a single larva in dorsal (left panel) and ventral views, the latter is depicted at two different magnifications. Scale bar, 500 µm; except **c, f** and **i**, 250 µm.

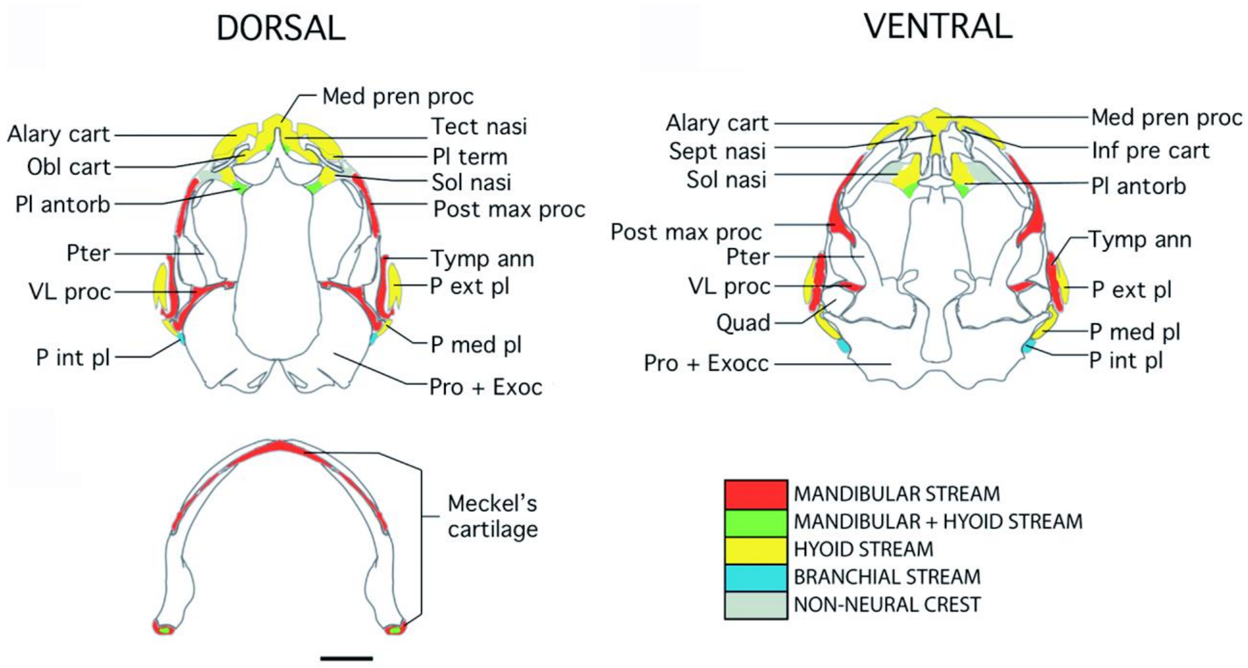

Supplementary Figure 2–Cranial neural crest (CNC) derivation of cartilages in the post-metamorphic skull of *Xenopus laevis* (NF Stage 66 + 1 month). Cartilages are shaded according to CNC stream(s); bones are unshaded. Abbreviations: Alary cart, alary cartilage; Exoc, exoccipital bone; Inf pre cart, inferior prenasal cartilage; Med pren proc, median prenasal process (of *septum nasi*); Obl cart, oblique cartilage; P ext pl, *pars externa plectri*; P int pl, *pars interna plectri*; Post max proc, posterior maxillary process; P med pl, *pars media plectri*; Pl antorb, *planum antorbitale*; Pl term, *planum terminale*; Pro, prootic bone; Pter, pterygoid process; Quad, *pars articularis* of the quadrate bone; Sept nasi, *septum nasi*; Sol nasi, *solum nasi*; Tect nasi, *tectum nasi*; Tymp ann, tympanic annulus; VL proc, ventrolateral process. Scale bar, 2 mm. Reproduced with permission from ref. 22.

Supplementary References

1. Gross, J. B. & Hanken, J. Use of fluorescent dextran conjugates as a long-term marker of osteogenic neural crest in frogs. *Dev. Dynam.* **230,** 100–106 (2004).
2. Sadaghiani, B. & Thiébaud C. H. Neural crest development in the *Xenopus laevis* embryo, studied by interspecific transplantation and scanning electron microscopy. *Dev. Biol.* **124,** 91–110 (1987).
3. Gross, J. B. & Hanken, J. Segmentation of the vertebrate skull: neural-crest derivation of adult cartilages in the clawed frog, *Xenopus laevis*. *Integ. Comp. Biol.* **48,** 681–696 (2008).
